# Global trends of suicidal thought, suicidal ideation, and self-harm during COVID-19 pandemic: a systematic review

**DOI:** 10.1186/s41935-022-00286-2

**Published:** 2022-06-04

**Authors:** S S Shobhana, K. G Raviraj

**Affiliations:** 1 Department of Forensic Medicine & Toxicology, St.Peter’s Medical College, Hospital and Research Institute, Hosur, Krishnagiri District, Tamil Nadu, 635130 India; 2 Department of Forensic Medicine & Toxicology, East Point College of Medical Sciences and Research Centre, Bangalore, India

**Keywords:** Suicidal thoughts, Suicidal ideation, Self-harm, Global scenario, COVID-19 pandemic

## Abstract

**Background:**

Suicide is one of the leading causes of death. The current systematic review is done to know the trend of suicidal thoughts, suicidal ideation, and self-harm during COVID-19 pandemic.

**Main text:**

The search was done by using PubMed, ScienceDirect, and Google Scholar databases. With the help of Mendeley portal, articles were retrieved on the basis of inclusion criteria like to know the risk factors, vulnerable group, complete article PDFs, prevention strategies, aims, results, and limitations. The shortlisted data from search was tabulated, and the PRISMA chart was framed based on the inclusion and exclusion criteria.

**Result:**

Sixteen studies that satisfied the inclusion criteria were organized and selected. The variables and global scenario were considered in databases. It has been noticed that trends of suicidal thoughts, suicidal ideation, and self-harm remains the same in some countries like Japan, whereas in some countries like Bangladesh and France, trends of suicides had increased during the pandemic period. The probable cause could be lockdown, social isolation, and stoppage of recreational activities.

**Conclusions:**

The trends of suicidal ideation, suicidal thoughts, and self-harm are more in vulnerable categories like health care professionals, university students, elderly individuals, and psychiatrically ill patients. In health care professional, it is due to the increased risk of contagion and watching deaths closely. In university students, it is due to the lack of recreational activities and social isolation. Among elderly, it is due to thinking themselves as overburden. The increase in suicidality in psychiatric ill patient admitted during COVID-19 pandemic is due to unknown cause.

## Background

Suicide is one of the leading causes of death globally, the World Health Organization had declared that with every suicide, there will be 20 suicide attempts (Klomek 2020). There is an increased risk of suicides, suicidal thoughts, and ideation with the onset of COVID-19 pandemic. (Pramukti et al. 2020), (Ammerman et al. 2021) With the onset of the novel coronavirus in China, the World Health Organization (WHO) had declared the novel coronavirus (COVID-19) outbreak as a global pandemic on March 11, 2020 (Sohrabi et al. 2020). Mortality and morbidity are increasing due to high infection rate of COVID-19 that had made governments of several countries to declare lockdown, compulsory wearing mask, social isolation, and social distancing norms. This had a vilest impact on psychological, social, and economic balance worldwide. The vulnerable groups especially are at increased risk of the impact, like health care professionals, as they are having an increased risk of suicidality compared to other occupational groups; they also suffer from an increase in workload (Galbraith et al. 2021) adolescents like those who are not going to schools or universities as they had to limit themselves to online classes; and they have negative effects with smoking and drinking (Nomura et al. 2021). An increase in incidence among psychiatric ill individuals are also noted (Berardelli et al. 2021) as well as an increase in suicide risk among working categories as many of them had lost jobs due to economic break down, low socio-economic sector, and among caregivers. There is an increase in depression, anxiety, insomnia, increase in fear of contagion, fear of losing loved ones, fear of transmitting infection to loved ones, and substance abuse. This had led to increase in chance of suicidal ideation, suicidal thoughts, and self-harm and had also raised requirements of mental health services. To deal with COVID-19 crisis, there must be integrated services in the treatment or handling the unattended suicidal ideation group of people. Strategies had to be planned to reduce suicides and psychosocial distress (Leaune et al. 2020). Several good things had also happened during lockdown along with negative psychological effects like enjoying work from home, spending more time with family, and relieve from fear of contagion (Sher 2020), (Every-Palmer et al. 2020). The current systematic review has been made to note the global trends of suicidal ideation, self-harm, and suicidal thoughts amidst the pandemic.

## Main text

**Fig. 1:**
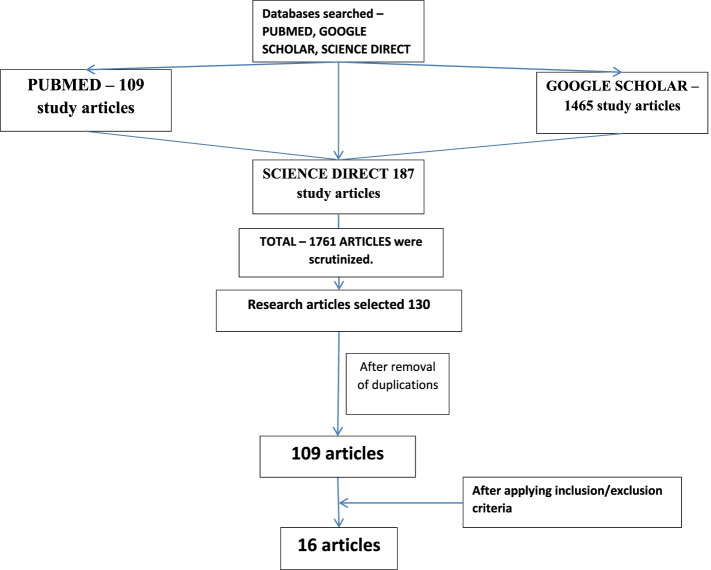
Flowchart displaying the methodology

This study was done on suicidal thoughts, suicidal ideation, and self-harm among the different sets of population during COVID-19 pandemic. Articles were searched by the keywords of suicidal deaths, suicidal thoughts, suicidal ideation, suicidal behavior, and self-harm. The articles with only abstracts without complete PDF and non-English articles were excluded. Articles preferably of 2021 were selected and global scenario was kept in mind while conducting the study. The data was last searched on 01 August 2021, databases searched were 109 articles from PubMed, 187 articles from ScienceDirect, and 1465 articles from Google Scholar. A manual search was conducted other than the abovementioned databases, and by typing keywords in Google chrome, other searching portals, and searching in some state and national journals were not prolific. It did not give satisfactory results like there were no complete article, and they were not in English language and did not match the inclusion criteria of the current study. Mendeley search engine and organizer was the reference manager software utilized for this purpose for the articles including research articles, reviews, case studies, correspondence letters, and letters to editors. Data was tabulated in Microsoft Excel by opening three separate sheets for each database. The articles that were included in study were only research articles with the availability of full articles, and those articles which satisfy the objectives and covariables like where strategies were given for preventions, aims were considered, country in which study was done was considered, and vulnerable population was studied along with risk factors. The articles were reviewed by two authors independently. Duplications were removed, and those articles satisfying the inclusion criteria were tabulated. To minimize the bias, it was reviewed by two authors independently. The flowchart was prepared following the PRISMA guidelines (Please see Fig. 1).

Criteria for study (inclusion criteria).

1. The study article included all the cases where there was mention of suicidal deaths, suicidal thoughts, suicidal ideation, suicidal behavior, and self-harm, either collectively or independently during COVID-19 pandemic.
2. Full article PDF, considering the vulnerable groups, prevention strategies, risk factors, year of publication with aims, results, and limitations were considered.

## Results

Out of 109 research articles searched, only 16 articles satisfied the inclusion criteria of the study. The remaining studies, even though were of good sources for this study, did not fully satisfy the criteria; hence, they were considered for discussion of objectives that were intended in the study. The results were tabulated, and limitations of certain studies are also noted in the table (Please see Table 1).

**Table 1:** Table representing the articles selected fulfilling the inclusion criteria

**SL NO**	**Author**	**Vulnerable Group**	**Risk Factors**	**Period**	**AIMS**	**PLACE**	**Results**	**Authors suggestions in tackling the risk factors observed in the study**
1	Cheung T et al. (Cheung et al. 2021)	Younger age	Anxiety, loss of employment, isolation, and male gender	2020	To examine the prevalence and corelates of suicidal ideation in 10 countries during pandemic, examining individual health belief association with suicidal ideation, and direction for its prevention	Eastern & western countries	Low suicidal ideation in UK & Brazil compared to Macau, more among young married male, with differential health belief. Association of face mask wearing had shown less suicidal ideation **Limitation of study:** Cross-sectional study cannot infer any causal relationship between variability and cannot be generalised **Prevention:** Joint international collaboration for suicide prevention strategies	Implementing more of online recreational activities, small-scale household production and supply for improving financial crisis
2	Kohls E, Baldofski S et al. (Kohls et al. 2021)	Students	Stress, loneliness, low social support	2021	Mental health status assessment with standardise measures	Germany	Study suggested university students are more prone for depression in Germany. Online intervention promotes the help seeking measures and it targeted on mental health **Limitation:** Standardized inferences could not be drawn due to large sample size **Prevention:** Online intervention support	Online psychotherapy and recreational activities like video chatting
3	Edith Hermosillo De La torre A et al. (Edith Hermosillo-de-la-Torre et al. 2021)	14–21 years Young adults and adolescents	Self-harm, intoxication	2021	Association between suicidal behavior with covariables like anxiety, depression, and drug use	Mexico	21% of students showed suicidal behavior, with increased incidence in female population, and those with depression, anxiety, and drug intake **Limitation:** Strategies to improve mental health had not been mentioned **Prevention:** Vaccine against Infection	Rehabilitation clubs to be made online
4	Silverio murillo A (Silverio-Murillo et al. 2021)	General population	Insomnia, stress, and anxiety suicides	2021	Internet search including insomnia, anxiety, depression, and suicides	Latin America	There is increase in anxiety and stress during pandemic and no change in depression and suicide **Limitation:** Internet search cannot be generalized among population **Prevention:** Income support by country	Online YOGA sections and meditations
5	Bruffaerts R (Bruffaerts et al. 2021 Mar)	Health care professional	Suicidal thought, behavior	2021	Cross-sectional survey of assessing association of suicidal thought and behavior among health care professional	Belgium	There were increase in prevalence of death wish, suicidal ideation, and suicide plan but no suicide attempts **Limitation:** Study did not assess the generalized suicidal thought and behavior but assessed during COVID-19 pandemic **Prevention:** Decrease in suicidal thoughts and behavior among those received social support	Shifts in working, to reduce stress, chatting with professional colleagues
6	D'Hondt F (D’Hondt et al. 2020)	College students	Social isolation	2020	Online survey among university students for assessing self-reported mental health, associated factor, and assess care-seeking during quarantine	France	Increase in the prevalence of mental health issue among students, to underline surveillance prevention and access care **Limitation:** Large sample size **Prevention:** Access care and prevention	Peer group online quiz, online library, online games
7	Pramukti E (Pramukti et al. 2020)	College students	Anxiety, suicidal thought, infection, and contraction	2020	Social media used to collect information about suicidal thought, anxiety, and ideation among Taiwan, Thailand and Indonesia	Taiwan, Thailand, Indonesia	Increase in suicidal thoughts and suicidal ideation among Taiwan students than Indonesia and Thailand students **Limitation:** Social media may not be used by everyone **Prevention:** Adequate support mechanism	Social media support to spread awareness, strengthening health care facilities
8	Rahman M (Rahman et al. 2021)	General population	Depression and anxiety	2021	Online assessment of suicidal behavior and ideation	Bangladesh	Increase in incidence among females, 33% cases showing suicidal risk, increase in incidence among divorced, widow, and low education statues **Limitation:** Online survey cannot be generalized **Prevention:** Awareness of COVID-19 pandemic, improving mental health status	Social workers to be trained and accessibility even to remote set up to be formulated by government and NGOs
9	Yom-Tov E (Yom-Tov et al. 2021)	Covid 19 Patient	Anosmia, ageusia	2021	Studying increase risk of suicidal ideation among anosmia and ageusia patients	USA	Effect of COVID-19 on senses will have a long-lasting effect and implication on patient well being **Limitation:** Self-diagnosis of disease **Prevention:** Special precaution during treatment and recovery	Awareness to be spread among public about Dos and Don’ts in Covid-19 pandemic, with the help of media
10	Mamum M (Mamun et al. 2021)	General Population	Fear of COVID-19, comorbidities, insomnia, suicidal ideation, and depression	2021	Cross-sectional survey about psychological effect by sociodemographic data, fear of COVID-19, insomnia, and comorbidities	Bangladesh	Increase in the prevalence of depression and suicidal ideation among young, females, comorbidities, infection, and cigarette smoking **Limitation:** Self-reported scale **Prevention:** Psychological support and good mental health services	Newspaper articles and lectures in media by doctors and psychotherapist
11	Daly Z (Daly et al. 2021)	General population	Suicides and self-harm	2021	Univariate and multivariate analysis of suicidality and self-harm with mental health issues	Canada	Increase incidence of suicide and self-harm among quarantine individual but less incidence among those quarantine after recent travel **Prevention:** Public health response to be mitigated to reduce mental health issues and suicidality	Online counseling
12	Staples L (Staples et al. 2020)	General population	Depression and anxiety	2021	Analysing use of digital mental health services prior COVID-19 and during COVID-19 with a questionnaire-based study	Australia	Increase in the number of users of digital mental health services during first week of COVID-19 and subsequently **Limitation:** Large section of society, economy and long run COVID-19 are to be considered **Prevention:** Adopting helpful strategies to improve mental health	Training junior doctors and nursing staff, so that counseling is easily available for everyone
13	Every palmar S (Every-Palmer et al. 2020)	Adults	Lockdown	2021	To access psychological wellbeing with lockdown effect, reducing job opportunities, less recreation activities by means of Kessler Psychological Distress Scale (K10), the GAD-7, and the Well-Being Index (WHO-5)	New Zealand	**Prevention:** Adequate provision of psychosocial support with similar priority to contact tracing, provision of personal protective equipment, and procurement of ventilators	Adequate medical aids
14	Shongwe M (Shongwe and Huang 2021)	Adult	Perceived stressors, prevalence of psychological distress, and suicidal ideation	2021	This study was a cross-sectional, population-based household telephone survey of 993 conveniently sampled adults (18 + years) from all the four administrative regions of Eswatini. COVID-19-related psychological distress was assessed using the Kessler 6-item Psychological Distress Scale (K	Eswatini	Increased risk for moderate/severe psychological distress **Limitation:** cannot be generalised as there may be non-telephone users **Prevention:** Government health policies to relive psychological distress	
15	Sahimi H (Sahimi et al. 2021)	Health care workers	Suicidal ideation and depression	2021	To investigate suicidal ideation in terms of the rate and associated factors in a sample of Malaysian healthcare workers during the early-phase of the COVID-19 pandemic	Malaysian	Increase in proportion of health care workers suicidal ideation and clinical depression **Limitation:** Small sample size, casualty of suicidal ideation not known, cannot be generalized **Prevention:** Identification and treatment of depression, early intervention	Regular meeting with peer group and prevention strategies among health care professionals
16	Maatouk I (Maatouk et al. 2021)	Adults	Job security, increased risk of infection, suicidal ideation, self-harm	2021	This cross-sectional study focuses on the social psychological correlates of self-harm and suicidal ideation during the COVID-19 (coronavirus disease 2019) outbreak in Lebanon, which is a country characterized by political and economic instability	Lebanon	Male and with low income had raised risk of suicidal ideation and self-harm. Political trust, religiosity, high-income and female group had less chances of suicidal ideation and self-harm **Limitation:** cannot be generalized **Prevention:** Need to ensure adequate access to mental health services to the general population amid the COVID-19 outbreak	Temporary small-scale production setups at house by family to improvise financial status, with government supports

## Discussion

Suicide accounts for 800,000 deaths globally which is quite a huge number among preventable causes of deaths. Identifying the factors and timely intervention will help in reducing this loss due to suicides. Quarantine, unemployment, (Nomura et al. 2021) economic crisis, and lack of international trades following pandemic had led to physical and mental imbalance among people. Unknown psychological distress had been raised due to the stigma and discrimination. Mass quarantine and stay at home had increased the risk of suicides. There is an increase in incidences of suicides among children and adolescents as they are staying at home. There is an increased risk of suicides among psychiatric patients as they are away from treatment during pandemic and also those indulged in withdrawal symptoms of substance abuse, and there is loss of employment in 20–30% of population which also rises the suicide risk (Que et al. 2020).

The authors’ suggestion for the state of affair that was prevailing during pandemic is to start online counseling programs for psychological and mental strength. This would reduce the risk of loneliness among those who led an isolated life during pandemic situation. This step should be brought out by the government with the help of health sectors.

Suicidal grief is particularly challenging and gives rise to complicated grief. Complicated grief usually follows the acute grief process in which bereavement reaction is prolonged, causing distress and interfering with functioning. The bereaved family will feel not worth leaving without their departed member. They will have feeling of longing which does not abate with time. Complicated grief often leads to suicidal thoughts (Young et al. 2012). Social proximity to should be maintained by family and friends despite of physical distancing, and there must be empathetic and humane approach to maintain psychological balance. There should be some time or opportunity to recover from the damage caused by the COVID-19, and further improvement in health care system and other developments especially about improving mental health services should be prioritized so that lives can be saved from morbidity and mortality from mental health issues. This can be better monitored and handled by online support and psychotherapeutic interventions. Psychotherapy can be made accessible by digital mode or online mode in the pandemic situation, though it may be difficult and not so satisfactory for many psychotherapists, but has to be followed (Pinto et al. 2020), (Tullio et al. 2020). The authors’ perspective varies in certain situation, as in remote places where the online services are in-accessible, like in certain rural setups in developing countries; in such situations, there must be health care facilitators who are trained at least in minimal means of diagnosing the worsening psychological states of the individual. Facilities for referral services should be actively implicated in these extreme situations.

Nationwide lockdown was announced in UK on 23rd March 2020 to stay at home, isolation, and social distancing for the wellbeing and safety of population. Public health measures were taken for physical wellbeing, but for mental wellbeing, adequate understanding must be staged (O’connor et al.^ 2020^).

The information about “suicidal ideation” prevalence in different countries is lacking. In a multinational cross-sectional observational study of the prevalence of suicidal ideation, the possibility of suicide pandemic was suspected as there is a global economic crisis. Cases of suicidal ideation were more noticed in Macau compared to the UK and Brazil probably due to increase in migrant population, less access for sanitizer, overcrowding, social isolation, and lack of PPEs. In some countries, there is low welfare support from government. The younger age group individuals are more severely affected as they are psychologically disturbed by lockdown, more infection of COVID-19, and online class with lack of entertainment. The study had given strategies for the prevention of suicides in the countries, by giving importance to telemedicine and formulating public health/mental health specialist and text line support which will mitigate the problems of isolation and anxiety. Study also involved high-income countries like Macau where the incidences of suicides are high. International collaboration of mental health care services to be formulated to avoid suicide risk and to frame strategies (Cheung et al. 2021).

A cross-sectional and anonymous survey had been conducted on university students, Germany, during the month of July and August 2020 to assess mental health status and standardized measures like depressive symptoms, alcohol intake, and drug intake. Students were asked about psychosocial effect of lockdown and regulation measures. Patient health questionnaire-9 was formulated which had showed 14.5% of them suffering from suicidal thoughts (Kohls et al. 2021). The authors suggestion for the student group who are suffering from lack of recreational activities, suffering loneliness, and long online classes which had drastically changed their mindset and mental status. In this situation, government should implement some online programs of recreational activities like online YOGA classes, meditation programs, online conduction of quiz programs, online clubs for academic purposes for children to reduce their boredom, and suicidal ideation due to loss of bereaved family members.

In a letter written to the editor of *Asian Journal of Psychiatry*, they had described about a family who committed suicide following the loss of the elder one in family due to COVID-19 infection in Iran. It shows the survivor perception of death following the loss of their dear one in the family to cope up with grief and loss. Quality care program and communication can avoid the untoward situation (Pirnia et al. 2020).

A study conducted among 8033 Mexican adolescent population after taking informed consent, 51% were females and 49% were males of age ranging between14 and 21 years. Questionnaire were collected from Google forms. The main outcome of the study was suicidal behavior. The negative effect of COVID-19 pandemic had increased the suicidal behavior, this is also secondary for social distancing and confinement. Suicide and suicidal behavior are a global issue and are two of the preventable causes of death which increase during pandemic and outbreak. Suicides are the second leading cause of death in youngsters, and it had been noticed one million deaths per year globally. Suicide attempts are more in females, but suicide deaths are more in males (Edith Hermosillo-de-la-Torre et al. 2021).

A questionnaire-based study was done in 449 people of Peru to know fear of COVID-19 among them, and it had been found that fear was more among slum dwellers and stable workers due to loss of income and loss of job. The study concluded with prevention strategies by the government to underprivileged individual and provide mental health support (Tullio et al. 2020).

In a review done to know the suicide risk in India during COVID-19 pandemic where quarantine, lockdown, social distancing, and alcohol withdrawal were more among population, several strategies were suggested in the study by the support of media to provide information judiciously and take help from political leaders, religious leaders, and celebrities in providing information in a positive note, provision for mental health, and treatments among alcohol addicts (Ganesan et al. 2021).

The authors had experienced increased incidences of suicides among the alcohol addicts and some drug addicts in the pandemic situation, as lockdown had stopped the accessibility for alcohol and drugs which were accessible prior to lockdown. Drug withdrawal symptoms are also more which in extreme situation leads to commission of suicides among the addicts. Increase training among the junior medical staff in psychiatric counseling can be incorporated in handling the current condition, so that health services are available for all.

Mobility restriction will reduce the risk of viral transmission, but it has increased the risk of developing stress, income, unemployment, and anxiety. A study had conveyed stay at home will reduce spread of infection. Apart from this, it increases the mental health status due to extended time spent with household members which decrease commuting-related stress. The study was done in 11 Latin American countries where they had related mental health issue which improved with income support provided by legislation. They had used Google trend data to assess mental health in relation to COVID-19 lockdown (Silverio-Murillo et al. 2021).

Suicides account for 2.2% all years of life lost worldwide. A systematic review and metanalysis was done to know about suicide, self-harm, and thought of suicide. There is little evidence of epidemic on the suicides. However, incidence among elderly was more in Hong Kong in SARS epidemic, but incidence in Japan remain unaltered among youngsters in COVID-19 pandemic (Rogers et al. 2021). As a second objective metanalysis showed increase in prevalence of self-harm over a time period. In another study done in Japan, it also showed less increase in suicidality following COVID-19 pandemic (Anzai et al. 2021).

In a cross-sectional survey among healthcare professional to assess the 30-day suicidal thoughts and behavior among health care professionals, it showed an increased prevalence of death wish, suicidal ideation, and suicide plans, but no suicide attempts. The survey had showed that the status of the mind during COVID-19 pandemic is different which cannot be generalized (Mortier et al. 2021).

In a cross-sectional survey done as an online survey of questionnaire-based study showing relationship of depression and anxiety among college students in Texas University during pandemic, there was increase in anxiety and stress among college students during COVID-19 pandemic (Wang et al. 2020).

The authors suggestion for health care professionals are reducing working hour, by increasing shifts, which will give them break from monotony. Regular meeting up with inhouse professional colleagues. There should be facilities made for video chatting with family members and dear ones, so that it will reduce the pressure among health care professionals.

Along with general population, university students were affected during quarantine, and the mental health of the young adults is always a concern globally. A study was done in France among university students by an online survey to know mental health status during quarantine, and the results showed more concerns were suicidal thought, severe anxiety, depression, and perceived stress among students during COVID-19 pandemic quarantine (D’Hondt et al. 2020).

COVID-19 is not only a challenge to infectious disease medicine but for mental healthcare. Concern on psychological, neurological, and social impact of disease is present. There is an increase in the prevalence of suicidal thoughts, self-harm, and abuse in the UK with COVID-19 pandemic. There was an increase in the incidence of abuse as there was a lack of support due to social distancing and stay at home policies. Economic deprivation and unemployment were the additional factors affecting it. The study includes a Patient Health Questionnaire about self-harm, abuse, suicidal thought, and ideation at least one occasion, and demographic data was also collected. The results had shown an increase in psychological and physical abuse with an increase in suicidal thoughts and self-harm during the first month of lockdown (Iob et al. 2020).

The authors’ suggestion to solve the problems of unemployment by sitting at home, by starting homemade masks, clothes, basic necessities of life like sanitizers, tissues, soaps, paper bags, PPEs, and others. The government should make facilities of distribution, money management, and economic support for the productions. This will solve the temporary unemployment issues and keep people engage in activities.

A cross-country comparative study was done in Thailand, Taiwan, and Indonesia by using social media like Facebook, WhatsApp, and Line to know the suicidal thoughts and increase of fear of spread of infection and anxiety, and it showed that Taiwan students are more affected compared to Indonesia and Thailand (Pramukti et al. 2020).

Italy is among the countries which is worst affected by COVID-19 pandemic. The lockdown was initiated to limit the regular lifestyle activity and trip for necessary activity, work, and health emergency. The current study was done in public psychiatry clinic, and patients were grouped into two groups: one group admitted before COVID-19 pandemic and another group during pandemic. The results showed an increase in suicidal thoughts and ideation more in group admitted during pandemic and requires proper protective measures (Berardelli et al. 2021).

A cross-sectional online survey study was done in Bangladesh of 18 years and older population by an anonymous questionnaire which was prepared to assess depression and anxiety. Logistic regression analysis and Pearson correlation analysis were performed for the variables. The results showed that the incidence was common among women, divorced, and widowed, more among low education, with fear of contracting infection and those affected by COVID-19 infection. The study had proposed awareness about COVID-19 to prevent suicide risk (Mamun et al. 2021).

A study was done in Canada during the first wave of COVID-19 pandemic for 18 years and older individual about mental health status among quarantine due to various reasons like those symptoms of COVID-19; those in contact with infection cases, household, and self-isolate; and travel-restricted individual. Univariate and multivariate regression used to explore relationship of interest was used to compare suicidal and self-harm with mental health status for those reported and those who did not report during quarantine. Results had shown an increase in suicidal ideation and self-harm among those who has been quarantined; however, those who had quarantined following the recent travel had shown less involvement in suicide and self-harm risk. The public health response to be mitigated to reduce mental health issues and suicidality (Daly et al. 2021).

In a research article done in China where frontline nurses were assessed of their mental health during COVID-19, based on a questionnaire prepared to know about depression, anxiety, somatic disorder, and suicidal ideation. Demographic, stress, and support variables were entered into logistic regression analysis to identify the impact factors. The study analyzed poor mental health among the nurses during COVID-19 pandemic as they are frontline workers, and they have extreme physical and psychological stress during duty. They had increase chances of infection along with depression, insomnia, anxiety, stigma, and frustration (Hong et al. 2021).

Social networking like Facebook and other media were used to express the suicidal ideation or to write suicide note especially by young adults. They were reluctant to share information or problems with family or the physician. The careful watch on social media and networking can protect the lives (Islam et al. 2021).

A study was done among the pregnant women in the USA to know about mental health. It had showed increase in depression and anxiety among pregnant women, more so among the women who had canceled their appointments with doctors and more among those who had experienced death in the family (Liu et al. 2021).

The authors’ perspective in case of pregnant ladies is to increase services of telemedicine and regular online counseling about Do’s and Don’ts in pandemic and improving the mental health among the women as they will be having fear of contagion.

In a study done among pediatric emergency department between age group of 11 and 21, it had shown that increase in suicidal ideation and attempts is high during the year 2020 compared to 2019, suggesting increase attention to be given to the youth to prevent them from committing suicides (Hill et al. 2021).

A study was done among 4527 Norwegian population by using social media about suicidal attempts, suicidal thoughts, and alcohol intake as a factor in COVID-19 pandemic. The results had shown an increase in risk of alcohol intake, suicidal attempts, and thoughts as risk factors (Bonsaksen et al. 2021).

## Conclusions

One of the most common causes of death is suicide, especially among young individuals. The current study is done to know the trends of various variables related to suicides like suicidal behavior, suicidal thoughts, completed suicides, and self-harm during COVID-19 pandemic. It had reflected that in some countries, there is increase in trends of suicides whereas in some, there is no change in the statistics of suicide rate during pandemic. Age group which is at risk had not been specified in the majority of studies; in some studies, it had been mentioned that 11 to 21 years and university students are most vulnerable. The gender wise distribution was also not specific in majority of studies. The limitation in the current study results cannot be generalized to all countries due to changing trends. The recommendation that can be drawn out of current review is to improve mental health status among people, especially vulnerable population like health care professionals, who are in constant touch with contagious people, with lot of mental worries to cope up with increase in case load and closely watching the death of the people, and students who are also more vulnerable from this current state of isolation and social distancing norms and with subsequent threats of increase in cases as well as the pregnant ladies as there will be fear of contagion, and psychiatric ill patient as there is increase in suicidal ideation among them, due to lack or discontinuity in the treatment resulting in withdrawal in substance abuse cases. Promotion of vaccination and making it mandate is the best policy to control the pandemic which in turn reduces the risk of morbidity and mortality and in turn sense of suicide thoughts are reduced. Regular follow-up and proper counseling also provide strength to mental health.

## Data Availability

Yes.
